# Recent advances in the search of BCRP- and dual P-gp/BCRP-based multidrug resistance modulators

**DOI:** 10.20517/cdr.2019.31

**Published:** 2019-09-19

**Authors:** Silvia Dei, Laura Braconi, Maria Novella Romanelli, Elisabetta Teodori

**Affiliations:** Department of Neuroscience, Psychology, Drug Research and Child’s Health - Section of Pharmaceutical and Nutraceutical Sciences, University of Florence, via Ugo Schiff 6, Sesto Fiorentino (FI) 50019, Italy.

**Keywords:** Cancer, multidrug resistance, multidrug resistance modulators, ATP-binding cassette transporter inhibitors, P-glycoprotein, multidrug resistance-associated proteins, breast cancer resistance protein

## Abstract

The development of multidrug resistance (MDR) is one of the major challenges to the success of chemotherapy treatment of cancer. This phenomenon is often associated with the overexpression of the ATP-binding cassette (ABC) transporters P-gp (P-glycoprotein, ABCB1), multidrug resistance-associated protein 1, ABCC1 and breast cancer resistance protein, ABCG2 (BCRP). These transporters are constitutively expressed in many tissues playing relevant protective roles by the regulation of the permeability of biological membranes, but they are also overexpressed in malignant tissues. P-gp is the first efflux transporter discovered to be involved in cancer drug resistance, and over the years, inhibitors of this pump have been disclosed to administer them in combination with chemotherapeutic agents. Three generations of inhibitors of P-gp have been examined in preclinical and clinical studies; however, these trials have largely failed to demonstrate that coadministration of pump inhibitors elicits an improvement in therapeutic efficacy of antitumor agents, although some of the latest compounds show better results. Therefore, new and innovative strategies, such as the fallback to natural products and the discover of dual activity ligands emerged as new perspectives. BCRP is the most recently ABC protein identified to be involved in multidrug resistance. It is overexpressed in several haematological and solid tumours together with P-gp, threatening the therapeutic effectiveness of different chemotherapeutic drugs. The chemistry of recently described BCRP inhibitors and dual P-gp/BCRP inhibitors, as well as their preliminary pharmacological evaluation are discussed, and the most recent advances concerning these kinds of MDR modulators are reviewed.

## Introduction

The resistance of cancer cells to antineoplastic agents represents a substantial limitation to effective chemotherapy. This chemotherapeutic failure can be produced both by intrinsic and acquired resistance. Intrinsic or primary drug resistance occurs among malignant tumor cells which present some inherent characteristics of resistance to anticancer drugs since the beginning of therapy. This kind of resistance can be present also in a small population of tumor cells such as the cancer stem cells^[1]^. Acquired or secondary drug resistance instead is developed during treatment of tumors that were initially sensitive to drug treatment^[2]^. As an example, non-small cell lung cancer or pancreatic cancer are intrinsically resistant to anticancer drugs, while in breast cancer and leukemia, drug resistance can be acquired after treatment with drugs to which the cells were originally sensitive^[3]^.

Multidrug resistance (MDR) is a kind of acquired drug resistance of cancer cells, but also microorganisms, to a variety of chemotherapeutic drugs that are structurally and mechanistically unrelated^[4,5]^, even to drugs to which they have not been exposed previously^[6]^. Once MDR is acquired, the anti-cancer effects of chemotherapeutic drugs decrease. Cancer cells that are originally sensitive to a single anti-cancer drug later become resistant to multiple anti-cancer drugs.

MDR is a complex phenomenon that can result from several unrelated biochemical mechanisms, such as increased drug efflux and decreased drug influx, but also sequestration of anticancer drugs in lysosomes as well as in intracellular organelles and intercellular vesicles, drug inactivation or lack of activation, increased drug metabolism or detoxification, mutations in drug target, activation of survival responses, evasion of apoptosis, cancer stem cell regulation, miRNA regulation, hypoxia induction, increased DNA repair and epigenetic changes^[7-11]^. Some of these mechanisms may coexist, rendering the cell refractory to treatment with drugs acting on a single target.

The MDR phenotype is often linked to the overexpression of transmembrane efflux transporters. In fact, the most widely implicated and studied mechanism of MDR is that resulting from altered cell membrane transport^[12]^. This kind of MDR is due to a lower intracellular drug concentration, associated with accelerated efflux of the chemotherapeutic drug as a consequence of the overexpression of a number of integral membrane transporters that act as extrusion pumps. Several families of efflux pumps, that can use a variety of energy sources, are present in mammals and micro-organisms^[5,13]^. Among these, the most important and best characterized family in humans is the ATP-binding cassette (ABC) superfamily of efflux transporters, that use ATP as energy source^[14-16]^. Overexpression of ABC transporters, frequently detected in human solid and hematologic cancers, is a marker for drug resistance and decreased patient survival^[17]^; moreover, high levels of ABC transporters can be found also in cancer stem cells^[18]^.

The human ABC transporters consist of 49 members that are classified into seven subfamilies designated ABC-A to ABC-G according to the similarity of their amino acid sequences^[19]^. These proteins are overexpressed in cancer cells, but they are also present in several important tissues where they play a physiological role. In fact, they have been found in the epithelium cells at blood-brain barrier (BBB), the intestinal epithelium, the biliary canalicular membrane of hepatocytes and the proximal tubules of kidney, where they catalyze the transport of a large variety of structurally diverse compounds across cellular membranes by regulating the secretion of lipophilic molecules and the extrusion of xenobiotics that entered the organism^[20,21]^. Some ABC transporters are also responsible for the homeostasis of endogenous agents, and people carrying defected ABC genes may be more susceptible to specific diseases such as the Tangier’s disease, Stargardt’s disease, cystic fibrosis and adrenoleukodystrophy^[22,23]^.

The most important and best-known transporters belonging to the ATP-Binding Cassette superfamily in humans are P-glycoprotein (P-gp/MDR1/ABCB1)^[24-26]^, multidrug resistance protein 1 (MRP1/ABCC1)^[27,28]^ and breast cancer resistant protein (BCRP/ABCG2/MXR)^[29-31]^.

**Figure 1:**
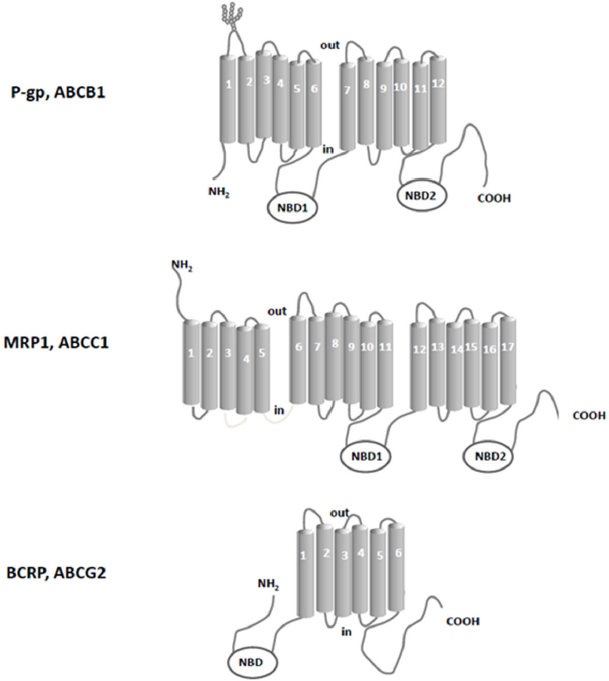
Schematic picture of the secondary structure of the ABC family drug transporters P-gp, MRP1 and BCRP. NBD: nucleotide binding domains

These three proteins, as it is for most ABC transporters, are composed of two domains: the transmembrane domain (TMD) and the nucleotide binding domain (NBD), which binds ATP. The NBD is located within cytoplasm; ATP binding and hydrolysis supply the energy for the transport of substrates across the membrane^[32]^. The TMD instead crosses the membrane and is composed of a number of transmembrane sequences, putative alfa-helices, separated by hydrophilic loops. The hydrophobic TMDs recognize and translocate a broad variety of substrates upon conformational changes, determining the characteristics of transported substrates. In Figure 1, the secondary structures of the ABC family drug transporters P-gp, MRP1 and BCRP are depicted.

Since the carriers must pump transported substrates against a chemical gradient, ATP hydrolysis represents the driving force, and the transmembrane domain must switch between outward- and inward-facing conformations, a mechanism already suggested by Jardetzky^[34]^ in 1966. The conformational switch of the membrane domain which is responsible for the alternating opening is driven by the binding of transport substrate and ATP magnesium salt, followed by ATP hydrolysis.

Several models have been proposed for the efflux mechanism, as the “alternating site” model^[35]^, the “switch” model^[36]^, and the “constant contact” model^[37]^, based on the biochemical and structural data obtained by multidrug resistance pumps of prokaryotes (Sav1866 from Staphylococcus aureus^[38]^, MsbA in gram-negative bacteria^[39]^) and eukaryotes (P-gp^[40,41]^, MRP1^[42]^ and TAP^[43]^). Although these models differ with respect to some of the details of the mechanism, they share some essential steps, such as ATP-dependent NBD dimerization and the switching of the TMD between outward- and inward-facing conformations.

**Figure 2:**
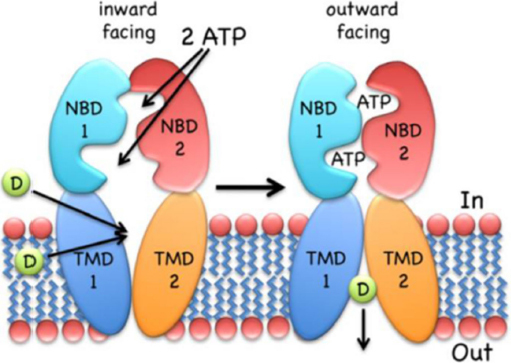
The inward-facing exporter binds substrate (D, drug) from the cytoplasm or the inner leaflet of the bilayer. After binding two molecules of MgATP, the nucleotide-binding domains (NBDs) dimerize and switch the transmembrane domain (TMDs) from the inward- to the outward-facing conformation, followed by the release of the drug to the extracellular environment. ATP hydrolysis, ADP/Pi release and NBD dissociation reset the transporter to the inward-facing conformation. The figure is quoted with permission from Wilkens^[47]^.

Drug translocation starts from the “apo” or ground state of the transporter; the first steps include substrate binding to the TMD and Mg-ATP binding (two molecules) to the NBDs. Substrates seem to bind at the high-affinity site within the TMDs, even if there can be changes of transporter structures at different stages. These bonds induce NBD dimerization and formation of the so called “ATP sandwich”, and the switch of the TMDs from the high-affinity inward conformation to the low-affinity outward conformation^[44,45]^, which releases the substrate out of the cell^[46]^. ATP hydrolysis and ADP/Pi release elicit NBD dissociation and reset the ground state of the transporter for the next cycle. Details and order of the steps depend, to some extent, on the transporter type; more information can be found in the paper by Wilkens^[47]^. In Figure 2 a scheme of the mechanism of ABC exporters is reported.

P-gp/ABCB1/MDR1, a 170 kDa glycoprotein, was the first ABC efflux transporter found to be responsible for the sensitivity of cells to chemotherapeutic agents^[48]^. P-gp isoforms are encoded by a small family of closely-related genes in humans (MDR1, MDR2/3)^[49]^, although only MDR1 is involved in multidrug resistance^[50]^. Human MDR1 is expressed as a single polypeptide consisting of 1280 amino acid residues that are organised in two domains of 610 amino acids, joined by a short 60 aa segment termed the linker region^[51,52]^. As most ABC efflux transporters, P-glycoprotein consists of two N-terminal TMDs and two C-terminal NBDs; each TMD contains six transmembrane segments (-helices). P-gp transports neutral and positively charged molecules: amphipathic compounds both in their unmodified form and as conjugates, lipid soluble compounds (molecular weights in the range of 300 to 1000) and compounds with aromatic rings and a positive charge at physiological pH^[53]^. On theses bases, it is clear that P-gp can transport many endogenous compounds such as steroid hormones, lipids, peptides and small cytokines^[54]^, but also a broad range of therapeutic drugs including anticancer drugs (e.g., vinca alkaloids, anthracyclines and taxanes), analgesics, antihistamines, antibiotics, antivirals, cardiac glycosides, calcium-channel blockers, calmodulin inhibitors and immunosuppressive agents^[55,56]^.

The second member of ABC efflux transporter revealed to confer MDR was MRP1/ABCC1^[57,58]^, which was over-expressed in cancer cells whose P-gp levels were not increased^[59,60]^. MRP1, a 190-kDa glycosylated polypeptide, is constituted by 1531 amino acids and is composed of three TMDs (17 transmembrane helices), two NBDs and one intracellular linker region. Like P-gp, MRP1 can confer the resistance to many chemotherapeutic agents; one difference between MRP1 and P-gp specificity for substrates is that taxanes are poor substances for MRP1^[61]^. In addition, MRP1 transports many endogenous and exogenous compounds as such or conjugated to GSH, glucuronides, and sulphates^[62,63]^. Other members of the ABCC family were described, which includes ABCC2-6 and ABCC10-12^[64]^. It was recently reported that also overexpression of some of these sister proteins was associated with resistance to anticancer drugs^[65]^.

The last discovered ABC efflux transporter involved in MDR is BCRP/ABCG2/MXR^[66]^. Its overexpression was first observed in 1998 in the multidrug resistant human breast cancer cell line MCF-7/AdrVp^[66]^. BCRP belongs to the subfamily G of the ABC transporter superfamily^[67]^ and is a 72-kDa half-ABC transporter, consisting of 655 amino acids; it has one TMD with six alpha-helices at the C-terminal end and one NBD at the N-terminal end. Nevertheless, it has been shown that the half-transporter BCRP, through the formation of disulfide bonds, functions as homodimer or homooligomer^[68-70]^. More recent investigations suggested that the transporter operates as a homotetramer^[71]^. BCRP is physiologically expressed in the gastrointestinal tract, liver, kidney, brain, endothelium, mammary tissue, testis and placenta; moreover, overexpression of this transporter was reported in untreated solid tumors and in different types of leukemia^[72]^, where the pump exhibits a very broad specificity for substrates like P-gp and MRP1. In fact BCRP actively extrudes a broad range of endogenous and exogenous substrates across biological membranes, as sulfate conjugates, glutamated folates, porphyrins^[56]^, and anticancer drugs like imatinib, methotrexate, camptothecin derivatives and epipodophyllotoxins^[73]^.

The relationships between ABC efflux transporter expression and tumor drug responses or patient survival have been well established for many years, considering patients after exposure to a chemotherapeutic agent. First evidences were described evaluating P-gp and/or MRP1 expression^[17,74,75]^. Also BCRP expression was observed in many tumors, and some studies indicate that BCRP could be a predictor of survival in patients with leukemia or cancer^[76,77]^. Interestingly, BCRP was found to be significantly co-expressed with P-gp^[78]^.

On these bases, identification of inhibitors against ABC transporters, such as P-glycoprotein (P-gp/MDR1/ABCB1, or multidrug resistance-associated proteins (MRPs/ABCCs), and breast cancer protein (BCRP/ABCG2) has emerged as critical goal because their inhibition may improve both chemotherapeutic response and patient outcomes. In fact, since the discovery and the clarification of the mechanism of action of the first efflux pump, modulation of the functions of P-gp and sister proteins has been considered an approach for fighting this kind of MDR. MDR reversers, also defined chemosensitizers, are pump modulators that co-administered with antineoplastic agents, which are substrates of the transporters, could restore the efficacy of anticancer drugs in resistant cancer cells^[79,80]^. Therefore drugs possessing modulating properties have been and are actively being sought^[81,80]^.

Verapamil was the first compound described to be able to reverse drug resistance in leukemia cells^[82]^ exhibiting P-gp modulating activity. Verapamil and other well-known molecules (e.g., quinine, trifluoperazine, progesterone and cyclosporine A) constitute the first generation of P-gp modulators, which were combined with different anticancer drugs. Unfortunately, the first-generation inhibitors were biological active compounds and they were not specifically developed for the modulation of ABC transporters. Moreover, many of them were also substrates for P-gp; thus, the use of high doses of chemosensitizers needed to inhibit the activity of ABC transporter produced toxic side effects and showed only limited or no benefits^[83]^. However, in the last decades many P-gp modulators have been identified^[84,85]^ and classified in different generations of compounds.

The second generation of P-gp-dependent MDR inhibitors were specifically designed to reduce possible toxicities and obtain higher potency and specificity by modifying the structures of P-gp modulators belonging to the first generation. In this way R-verapamil^[86]^, VX-710 (biricodar)^[87]^ and PSC-833 (valspodar^[88]^ were obtained. Indeed, these derivatives inhibit the function of P-gp, and do not exhibit the original activity of their precursors. Unfortunately, co-administration of the MDR modulators with anticancer drugs elicited pharmacokinetic interactions and altered the absorption, distribution, metabolism and excretion (ADME) of anticancer drugs leading in general to increased toxicity^[89,90]^.

The third generation P-gp dependent MDR modulators such as laniquidar (R101933)^[91]^, ONT-093 (OC14-093)^[92]^, zosuquidar (LY335979)^[93]^, elacridar (GF120918 or GW120918)^[94]^ and tariquidar (XR9576)^[95]^ have a high affinity to ABC transporters at nanomolar concentrations and show a limited CYP3A inhibition^[96,97]^; therefore they exhibit almost no pharmacokinetic interactions with the combined chemotherapeutic drugs, and some of them have reached pre-clinical or clinical stages^[98,99]^. Unfortunately, although *in vitro* studies have confirmed their effect as MDR modulators, clinical trials suggested that the activities of anticancer drugs were not improved by co-administration of these compounds^[100,101]^, and none of them has been approved for therapy. Nevertheless, the concept that modulation of ABC efflux transporters may overcome MDR is still strong, and the search for new chemosensitizers is still ongoing. More recently, some evidences have demonstrated a MDR reversing activity by using natural products as flavonoids, curcuminoids, taccalonolides and terpenes; so there is a growing interest in exploring the use of natural components of foods/plants as P-gp modulators^[99]^. These natural derivatives, together with some surfactant as Cremaphor EL and Nonidet P40 are considered as the fourth generation of MDR reversal agents^[102]^.

In the meantime, an intriguing aspect that emerged from the studies was that the ABC transporters P-gp, MRPs and BCRP are often co-expressed in tumors and that they have an overlapped specificity for a variety of substrates^[103]^. Therefore, selective inhibition of one efflux transporter could be compensated by the remaining transporters, and in the last years many studies were devoted to evaluating the inhibitory activity of new derivatives on cells overexpressing different transporters.

In the last two decades, after the discovery of BCRP, many new molecules acting as BCRP-dependent MDR modulators were reported, but also in this field sound SARs are lacking. BCRP is overexpressed in several haematological and solid tumours together with P-gp^[78]^. Moreover, P-gp and BCRP are the two main ABC transporters placed at the BBB and reduce the ability to cross the BBB of many drugs that are substrates of these two proteins including chemotherapeutic agents.

On this basis, selective BCRP inhibitors and dual P-gp/BCRP ones are needed to define sound structure-activity relationships (SARs) and to discover new leads for the development of new molecules. In this paper, both selective BCRP inhibitors and dual P-gp/BCRP inhibitors reported in the literature in the last five years were analysed and reviewed. Chemical structures, SARs and reported pharmacological characterization were described.

## Flavonoids and chromone derivatives

Flavonoids are a group of naturally occurring polyphenolic compounds present in vegetables and fruits. These natural compounds are important constituents of human diet and are well known for their beneficial effects on health. A variety of biological activities have been attributed to flavonoids, these include anti-oxidant, anti-inflammatory, anti-mutagenic, anti-viral, and anti-allergic properties and these compounds may exert protective effects against various disease conditions including cardiovascular disease and cancer^[104]^. In the last decades a variety of flavonoids have been investigated for their capacity to modulate the P-gp and BCRP activity. Some polyphenols can overcome cancer chemotherapeutic resistance as multidrug reversal agents and the combination of flavonoids and chemotherapy seems to be an interesting approach for cancer treatment^[105-107]^. Recently, several naturally occurring flavonoids as well as few synthetic analogues have been reported to be good inhibitors of BCRP^[108-114]^.

Chromones are a class of natural compounds, characterized by the presence of 1,4-benzopyrane structure. Some functionalized chromones were described as highly active compounds as MDR modulators^[115]^.

**Figure 3:**
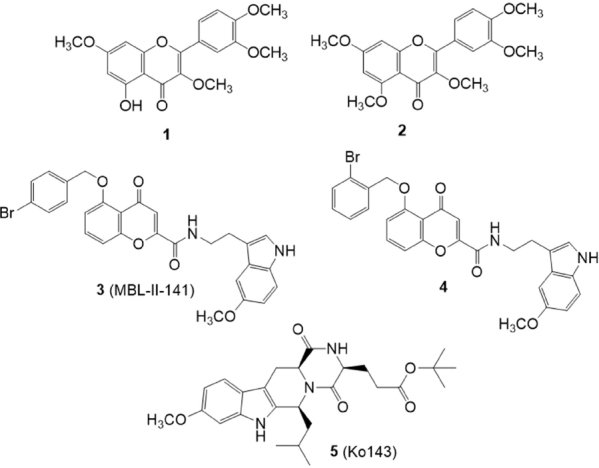
Flavonoids and chromone derivatives

Flavones bearing a methoxy group at position 3 of the pyranone ring [Figure 3] were recently identified as effective and selective BCRP inhibitors as they were able to increase the accumulation of Hoechst 33342 and pheophorbide A in MDCK BCRP cells^[116]^. Among these compounds, 1 was identified as a potent and selective BCRP inhibitor, while pentamethyl quercetin (2) was identified as a broad-spectrum inhibitor that showed substantial effect also on both P-gp and MRP1, evaluated by calcein AM assay on P-gp-overexpressing A2780 adr cells and MRP1-overexpressing 2008 MRP1 cells, respectively. Studies to investigate the substrate or inhibitor nature of compound 1 revealed that this 3-methoxy flavone was able to stimulate ATPase activity at low concentrations suggesting that it binds to the high affinity activating site of the transporter^[117]^. Otherwise, at higher concentrations it was able to lower the ATPase activity, suggesting that the affinities for the activating and inhibitory site are relatively close to each other.

The chromone derivative 3 (MBL-II-141)^[118,119]^ [Figure 3] is a potent and selective BCRP inhibitor endowed with excellent *in vivo* activity and very low toxicity. In 2016, a series of derivatives of compound 3 were synthesized and studied for their ability to inhibit the efflux of mitoxantrone (an anticancer drug and substrate of BCRP) in BCRP-transfected HEK293 cells and for their cytotoxicity^[120]^. Among the investigated compounds, derivative 4, bearing a 2-bromine atom on the benzyloxy moiety, was 3-fold more potent than 3 (EC_50_ = 0.086 M *versus* 0.26 M). It showed the same potency as Ko143 (5)^[121,122]^ (0.074 M), the most potent and selective BCRP inhibitor known today, but with the advantage of a lower intrinsic cytotoxicity.

## Chalcone derivatives

**Figure 4:**
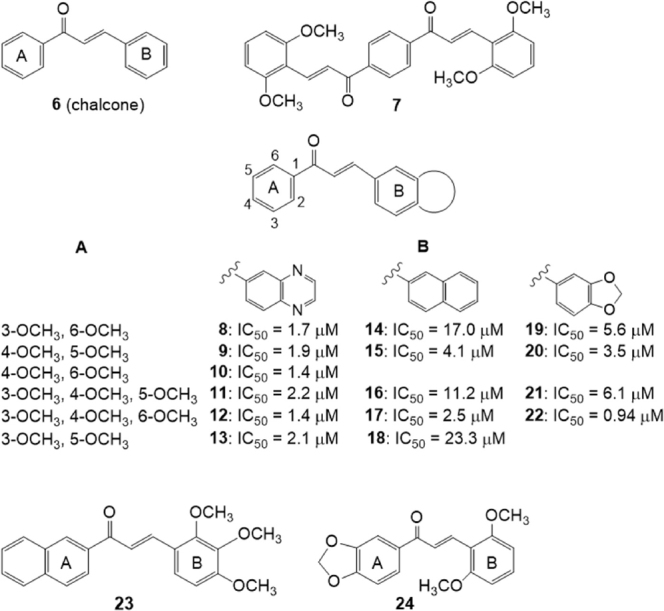
Chalcone derivatives

Derivatives of chalcone (6) [Figure 4] are a class of natural compounds, precursor of flavonoids, characterized by the presence of two phenyl moieties, A-ring and B-ring. They were shown to possess a variety of biological activities and several research groups have investigated chalcone derivatives for their P-gp and BCRP inhibitory effect^[123-126]^.

Differently substituted bis-chalcones have been investigated for their ability to inhibit mitoxantrone efflux from BCRP-transfected HEK293 cells^[127]^. The number and position of methoxy groups were critical for activity since compounds without this group had low efficiency; the best derivative was compound 7 (EC_50_ = 0.2 μM) with methoxy groups in position 2,6 of rings B of the two chalcone moieties. This compound, at the not cytotoxic 0.1 μM concentration, was able to sensitize to mitoxantrone toxicity the BCRP -transfected cells, as monitored by MTT assays, decreasing the IC_50_ (cytotoxic concentration for 50% cell survival) of mitoxantrone from 26.6 nM to 7.2 nM. Compound 7 can be considered a BCRP-selective inhibitor since it was not able to inhibit the P-glycoprotein-mediated mitoxantrone efflux in P-gp-transfected NIH-3T3 cells or the MRP1-mediated calcein efflux in MRP1-transfected HEK293 cells. The synergistic inhibitions observed for compound 7 when combined with a low concentration of chromone 3 (MBL-II-141) suggested that the two inhibitors may bind to distinct sites within BCRP. Compound 7 stimulated the BCRP basal ATPase activity while compound 3 was an inhibitor, suggesting different mechanisms of interaction.

A series of chalcones containing a bicyclic B-ring [Figure 4] were synthesized and tested as BCRP-mediated mitoxantrone efflux inhibitors on human fibroblast HEK293 cell lines transfected with BCRP (HEK293-ABCG2)^[128]^. The quinoxaline derivatives appeared more efficient than the 2-naphthyl or 3,4-methylenedioxyphenyl analogues. In all cases, the activity depends on the number and position of methoxy groups present on the phenyl A-ring and two or three methoxy groups produce a maximal inhibition. Molecular modeling indicated both electrostatic and steric positive contributions of these substituents.

The best quinoxaline derivatives (8-13), displayed IC_50_ values in the range 1.4-2.2 μM that are lower than those of the 2-naphthyl or 3,4-methylenedioxyphenyl analogues (compounds 14-18 and 19-22, respectively). In the 2-naphthyl or 3,4-methylenedioxyphenyl series, a higher potency was observed when the 2-naphthyl or 3,4-methylenedioxyphenyl group was shifted to the A-ring and methoxy substituents were shifted to the phenyl B-ring (23 and 24) indicating that the chalcones are functionally asymmetric^[128]^.

**Figure 5:**
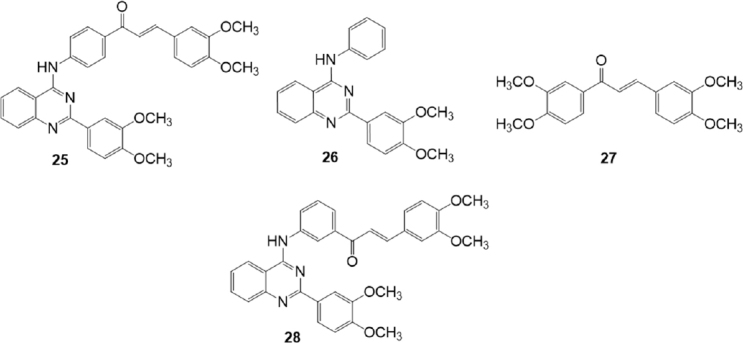
Chalcone quinazoline derivatives

In 2016, Kraege *et al.*^[129]^ investigated a series of 22 heterodimeric derivatives characterized by the presence of a quinazoline ring linked to a chalcone scaffold for their ability to inhibit BCRP, using the pheophorbide A assay on the MDCK II BCRP cell line [Figure 5]. The inhibitory activity toward P-gp and MRP1 was evaluated using the calcein accumulation assay on A2780 adr (P-gp) and H69AR (MRP1) cell lines, respectively. Structural features for inhibitory activity against BCRP were identified, like the presence of a quinazoline 2-phenyl ring bearing two methoxy groups and the presence of the 3,4-dimethoxy substituent on chalcone ring B, which caused an increase of inhibitory activity. The most potent BCRP inhibitor of this series was compound 25 (IC_50_ = 0.19 M) showed 25 fold higher inhibitory potency against BCRP than the individual building blocks, 2-(3,4-dimethoxyphenyl)-4-anilinoquinazoline (26) (IC_50_ = 4.09 μM)^[130]^ and (*E*)-1,3-bis(3,4-dimethoxyphenyl)prop-2-en-1-one (27) (IC_50_ = 5.12 M)^[131]^. Compound 25 was able to reverse MDR for the BCRP substrate SN-38 in the same concentration range and to the same extent as Ko143(5). Most compounds showed low inhibition of P-gp and no activity against MRP1, but some derivatives displayed a good activity also against P-gp, in some cases even higher than against BCRP. Compound 28, on which the acryloylphenyl residue of the chalcone is shifted in the meta position of ring A, showed IC_50_ value of 0.48 M on A2780 adr (P-gp) cell line and 0.60 M on MDCK II BCRP cell line; thus it can be considered as a dual P-gp/BCRP inhibitor^[129]^.

**Figure 6:**
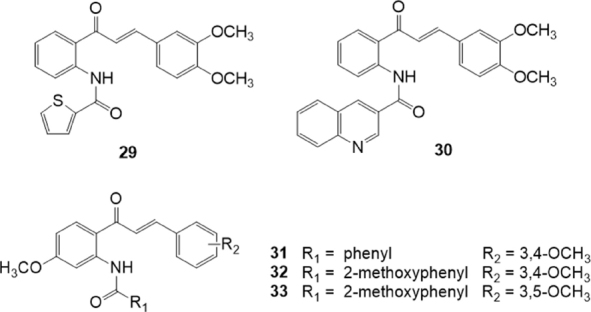
Acryloylphenylcarboxamide derivatives

Kraege *et al*.^[132]^, in 2016, reported another study on a series of 35 acryloylphenylcarboxamides obtained combining the chalcone scaffold with different substituted acid chlorides through an amide linker at position 2’, 3’, or 4’ on ring A of the chalcone moiety [Figure 6]. These compounds were investigated for their inhibitory activity on the BCRP overexpressing MDCK II BCRP cell line by using the pheophorbide A and Hoechst 33342 assays. The inhibitory activity against P-gp and MRP1 was studied on the P-gp overexpressing A2780 adr cells and MRP1 overexpressing H69AR cells, respectively, by using the calcein AM assay. The ortho position of the amide linker and the 3,4-dimethoxy groups on ring B have been found important for inhibitory activity. The most potent compound 29, which contains an unsubstituted thiophene ring, showed IC_50_ values of 0.60 M, in the pheophorbide A assay, and of 0.50 M in the Hoechst 33342 assay. This compound showed less affinity toward P-gp and was almost inactive against MRP1. Analogously, almost all compounds demonstrated some P-gp inhibition, but none exhibited significant activity against MRP1. Compound 30 showed the highest inhibitory potency against P-gp with IC_50_ = 0.494 M on A2780 adr (P-gp) cell line and also a quite good inhibitory activity against BCRP (IC_50_ = 0.971 M). For this reason, this compound can be considered as a dual P-gp/BCRP inhibitor.

In the same year, the same research group, reported another series of acryloylphenylcarboxamides [Figure 6] and of acryloylphenylcarboxylates with a 4’-methoxy group on ring A of the chalcone^[133]^. These compounds were investigated for their inhibitory activity on the BCRP overexpressing MDCK II BCRP cell line by using the pheophorbide A and Hoechst 33342 assays. The inhibitory activity against P-gp and MRP1 was studied by using the calcein AM assay on P-gp overexpressing A2780 adr and MRP1 overexpressing H69AR cells, respectively. The presence of a 4’-methoxy group on ring A of the chalcone moiety leads to an increased inhibitory activity toward BCRP in comparison with the unsubstituted series^[132]^. No inhibitory effect for MRP1 and weak affinity toward P-gp was observed, except for compounds bearing an additional methoxy group at the benzamide. Analyzing the structure-activity relationships, the presence of 3,4-dimethoxy groups on ring B of the chalcone was the most efficient pattern among those investigated on MDCK II BCRP cell line, and the replacement of the amide function with an ester decreased inhibitory effects toward BCRP. Compound 31 was the most potent derivative on BCRP with IC_50_ = 0.219 M, and it can be considered rather ABCG2 selective since on P-gp it showed an IC_50_ value of 1.13 M. On the contrary compounds 32 and 33 can be considered as a dual P-gp/BCRP inhibitors since they showed on BCRP IC_50_ values of 0.285 M and 0.595 M, respectively, and on P-gp IC_50_ values of 0.848 M and 0.250 M, respectively^[133]^.

**Figure 7:**
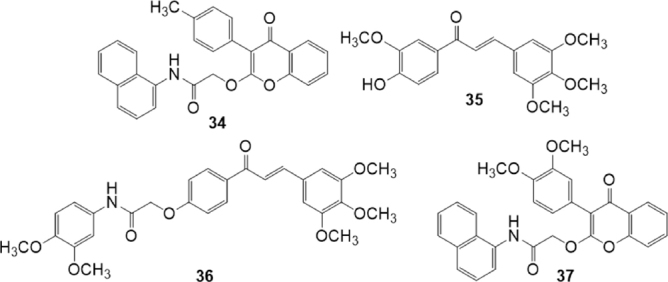
Chalcone and flavone derivatives

With the aim to develop selective as well as broad-spectrum inhibition of the transport proteins involved in MDR, Silbermann *et al*.^[134]^ reported in 2019 a study of the inhibitory effect of chalcone and flavone derivatives against P-gp, MRP1 and BCRP [Figure 7]. These compounds are chalcones variously substituted on rings A and B, and flavones with acetamido linker at position 2. The inhibitory activity of these compounds against P-gp, MRP1 and BCRP, was studied on P-gp overexpressing A2780 adr and MRP overexpressing H69AR cells, by using the calcein AM assay, and on BCRP-overexpressing MDCK II BCRP cells by using the pheophorbide A assay. Two specific inhibitors of P-gp and BCRP were found: compound 34 that inhibited P-gp-mediated calcein AM efflux with an IC_50_ value of 1.89 M, and compound 35 that was the most potent and selective ABCG2 inhibitor with an IC_50_ value of 1.97 M regarding BCRP-mediated pheophorbide A efflux. Compounds 36 and 37 were instead dual MRP1/BCRP and P-gp/BCRP inhibitors, respectively. The chalcone derivative 36 showed IC_50_ values of 12.5 M on MRP1 mediated calcein AM efflux and of 3.37 M on BCRP-mediated pheophorbide A efflux. The flavone derivative 37 showed IC_50_ values of 5.43 M on P-gp mediated calcein AM efflux, and of 3.75 M on BCRP-mediated pheophorbide A efflux^[134]^.

## Schizandrin derivatives

**Figure 8:**
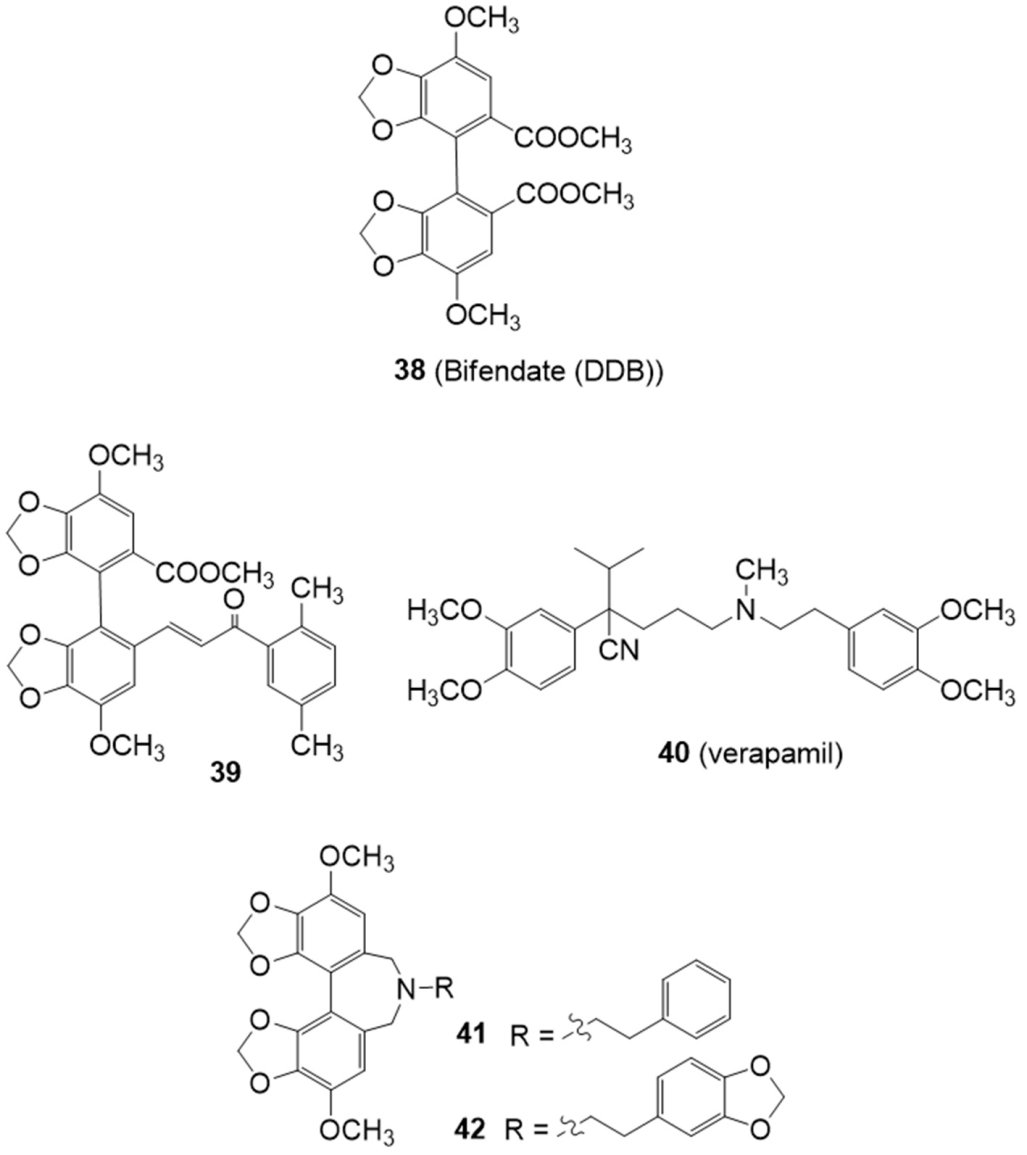
Schizandrin derivatives

Some synthetic derivatives of the natural compound schizandrin extracted from Schisandra chinensis, have the ability to restore drug sensitivity at non-toxic concentrations via direct interaction with P-gp. Bifendate (DDB) (38) [Figure 8] is an analogue of schizandrin C^[135]^, used for the treatment of chronic viral hepatitis B in China. Bifendate showed MDR reversal activity *in vitro* and *in vivo*, increasing intracellular accumulation of anticancer drugs and promoting cancer cell apoptosis through inhibition of P-gp^[136]^.

Bifendate-chalcone hybrids have been described as potent P-glycoprotein inhibitors with little intrinsic cytotoxicity^[137]^ [Figure 8]. On K562/A02 cells overexpressing P-gp (induced by adriamycin), compound 39 was able to increase the accumulation of rhodamine 123 more potently than the classical P-gp inhibitor verapamil (40) showing a very low intrinsic cytotoxicity (IC_50_ > 200 M). Furthermore, 39 showed no stimulation of the P-gp ATPase activity, suggesting it was not a P-gp substrate. In a following paper, the reversing activity of compound 39 on BCRP and MRP1 has been investigated on BCRP-transfected stable HEK293/BCRP cells and MRP1-transfected stable HEK293/MRP1 cells, respectively^[138]^. This compound exhibited little intrinsic cytotoxicity (IC_50_ > 100 M) against HEK293/BCRP cells and their corresponding BCRP-negative HEK293/VEC cells. It could reverse the BCRP-mediated efflux of the mitoxantrone with an activity almost comparable with the BCRP inhibitor Ko143 (5). Little inhibitory effect on multidrug resistance-associated protein 1 (MRP1) was showed in the adriamycin (a MRP1 substrate) accumulation test on MRP1-transfected stable HEK293/MRP1 cells. Thus compound 39 can be considered as a dual P-gp/BCRP inhibitor.

Gu *et al.*^[139]^ reported that a series of bifendate derivatives bearing dibenzo[c,e]azepine scaffold could reverse P-gp-mediated MDR by blocking drug efflux function in K562/A02 MDR cells [Figure 8]. The most potent compounds 41 and 42 reversed P-gp-mediated multidrug resistance (MDR) with an activity higher than bifendate (38) and verapamil (40). Compound 42 showed no stimulation on the P-gp ATPase activity, suggesting it is not a substrate of P-gp.

Moreover compounds 41 and 42 could markedly increase mitoxantrone accumulation in HEK293/BCRP cells via inhibiting BCRP efflux function with an inhibitory activity comparable to that of the classical BCRP inhibitor Ko143 5^[140]^. Compound 42 could not increase the MRP1 substrate adriamycin accumulation in MRP1-mediated MDR in MRP1-transfected stable HEK293 cells (HEK293/MRP1 cells), suggesting 42 may be considered a dual inhibitor of P-gp/BCRP.

## Curcumin derivatives

Curcumin is a polyphenolic natural product known for its properties as antioxidant, ant-inflammatory and antitumor agent. Its metabolites or constituents were found able to reverse the drug resistance in cells mediated by P-gp, MRP1 and BCRP, in particular^[141-143]^.

**Figure 9:**
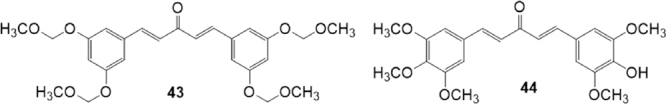
Curcumin derivatives

In 2017, Murakami *et al.*^[144]^ investigated a series of 24 synthetic curcumin analogues on the transport function of BCRP by using BCRP-overexpressing K562/BCRP cells [Figure 9]. Two curcumin analogues ^(43,44)^ were able to inhibit significantly the efflux of the BCRP substrates mitoxantrone and pheophorbide A from BCRP-overexpressing K562/BCRP cells. These compounds stimulated the ATPase activity of BCRP at nanomolar concentrations and inhibited the photolabeling of BCRP with iodoarylazidoprazosin, suggesting that they are able to inhibit the function of BCRP by directly interacting at the substrate-binding site.

## Quinazoline derivatives

**Figure 10:**
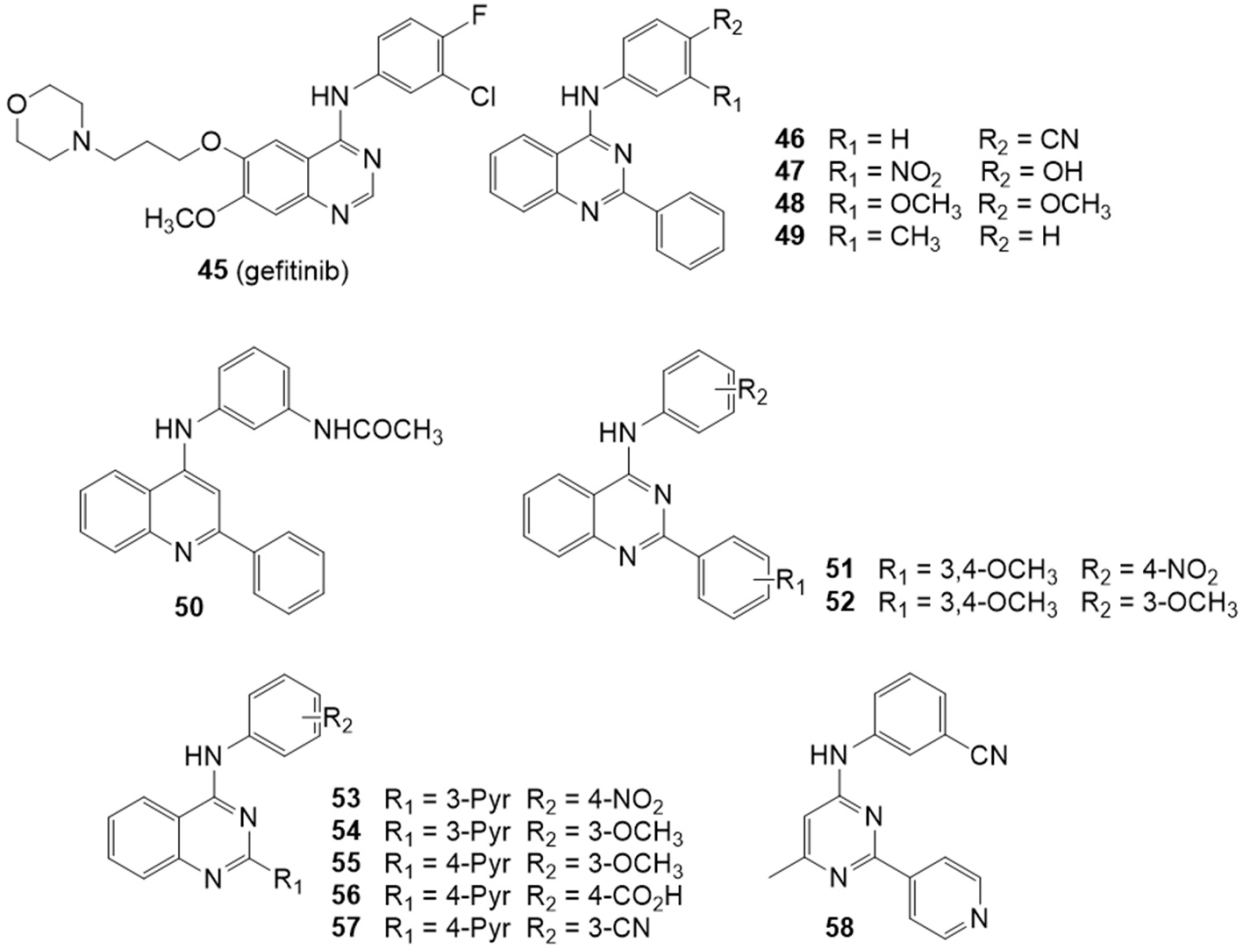
Quinazoline and quinoline derivatives

Recently, it was demonstrated that some tyrosine kinase inhibitors (TKIs) are able to inhibit P-gp or BCRP transporters. The quinazoline based TKI gefitinib (45) [Figure 10], in particular, has been reported to be a potent BCRP inhibitor^[145]^.

In 2016, Krapf *et al*.^[146]^ investigated several *meta* and *para* substituted 4-anilinoquinazolines for their inhibitory activity on BCRP using the Hoechst 33342 accumulation assay on the BCRP overexpressing MDCK II BCRP cell line [Figure 10]. The inhibitory activity on P-gp and MRP1 was investigated on the P-gp overexpressing cell line A2780 adr and the MRP1 overexpressing cell line H69AR respectively, by using the calcein AM accumulation assay. The compounds that showed the best inhibitory activity toward BCRP were characterized by the presence of hydroxy, cyano, nitro, acetamido, and fluoro groups at the 4-aniline scaffold. In the Hoechst 33342 accumulation assay toward the BCRP overexpressing MDCK II cell line, compounds 46 and 47 were the most potent inhibitors with a 3-fold higher potency than Ko143(5) (IC_50_ = 69.9, 80.0 and 221 nM, respectively). These compounds showed no inhibitory activity toward P-gp and MRP1 thus proving to be highly BCRP selective. Compounds 48 and 49 that showed on BCRP IC_50_ values of 0.152 and 1.150 M respectively, exhibited also some inhibitory activity toward P-gp (IC_50_ = 1.86 and 3.00 M, respectively) but no activity toward MRP1. Therefore, they can be considered dual P-gp/BCRP inhibitors.

Some quinoline analogues were also studied to evaluate the importance of the nitrogen atom at position 3 and the results showed that the presence of a nitrogen in this position is crucial for activity and selectivity toward BCRP^[146]^ [Figure 10]. In fact, as example, compound 50 showed a low inhibitory activity towards BCRP (IC_50_ = 1.63 M), a quite good activity on P-gp (IC_50_ = 0.66 M) and was able also to slightly inhibit the calcein AM efflux on the MRP1 overexpressing cell line H69AR (IC_50_ = 5.80 M).

The next year, Krapf *et al.*^[147]^ reported another series of BCRP modulators based on quinazoline scaffold [Figure 10]. These compounds are 2-phenyl-4-anilinoquinazolines with various substituents on the aromatic systems at position 2 and 4 of the quinazoline scaffold. The Hoechst 33342 accumulation assay was used to investigate their inhibitory activity on BCRP overexpressing MDCK II cell line; the activity on P-gp overexpressing A2780 adr cell line and MRP1 overexpressing H69AR cell line was studied using the calcein AM accumulation assay. This study allowed to identify several potent inhibitors possessing high selectivity toward BCRP as well as some potent dual inhibitors of P-gp/BCRP. On the contrary, only a few compounds showed a slight MRP1 inhibitory activity. The best features for high inhibitory activity on BCRP were the combinations of nitro with methoxy groups. In particular, compounds with 3,4-dimethoxy moieties and meta or para nitro substituents on the two aromatic systems, were found to be highly potent. The most potent compound was 51 that showed an IC_50_ value of 44.2 nM, that is five time lower than that of Ko143(5) (IC_50_ = 221 nM). Several compounds with two or more methoxy groups showed inhibitory activity toward P-gp. Compound 52, in the calcein AM accumulation assay on P-gp overexpressing A2780 adr cell line, was more potent than the standard reference cyclosporine A (IC_50_ =1.04 and 1.21 M, respectively); it displayed an IC_50_ value of 0.365 M on BCRP while was inactive on MRP1. Compounds 51 and 52 were further studied to define their mode of interaction with the BCRP substrate Hoechst 33342 which acts as fluorescent dye. A competitive binding mode was found for compound 51, while compound 52 was found to be a non-competitive inhibitor.

In the same year a series of 4-anilino-2-pyridylquinazolines containing also compounds with the quinazoline scaffold reduced to a 4-methylpyrimidine was reported^[148]^ [Figure 10]. The inhibitory activity towards BCRP was evaluated by the Hoechst 33342 accumulation assay using the MDCK II BCRP cell line. The activity on P-gp and MRP1 was investigated by the calcein AM assay using P-gp overexpressing cell line A2780 adr and MRP1 overexpressing cell line H69AR, respectively.

The most potent 3-pyridyl derivative 53 contained a *para* nitro-phenyl moiety and showed in the Hoechst 33342 accumulation assay an IC_50_ value of 64.1 nM, which is 3.5-fold lower than for the reference compound Ko143 (5) (IC_50_ = 227 nM). None compounds showed significant activity toward MRP1 while compounds with one or more methoxy groups at the phenyl ring showed activity toward P-gp as compounds 54 (IC_50_ = 4.78 M) and 55 (IC_50_ = 5.43 M). Interestingly, compound 56 with a 4-carboxy group at phenyl moiety showed the best P-gp inhibitory activity (IC_50_ = 3.67 M). Among the 4-methylpyrimidine derivatives, exemplified by 58, several compounds yielded good BCRP activity but none showed significant activity toward P-gp. In the Hoechst 33342 accumulation assay the 4-methylpyrimidine derivative 58 showed an inhibitory activity similar to the corresponding quinazoline analogue 57 (IC_50_ = 132 and 134 nM, respectively) demonstrating the high ligand efficacy of this scaffold)^[148]^.

## Tariquidar derivatives

**Figure 11:**
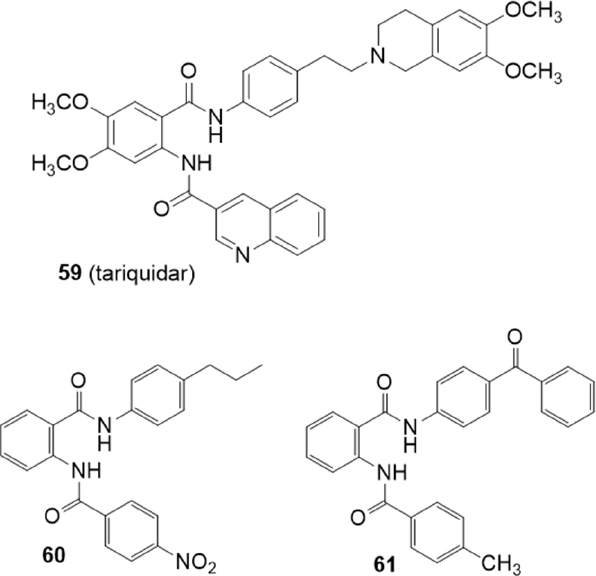
Tariquidar derivatives

Tariquidar (XR9576) (59) [Figure 11] is a third generation P-gp inhibitor that produces specific and effective inhibition of P-gp function at nanomolar concentrations^[149-150]^ and reached phase III clinical trials. However, no substantial benefits have been established due to inefficacy and toxic effects^[151]^. Tariquidar (59) showed dual inhibitory activity on P-gp than BCRP, being two times more selective in inhibiting P-gp than BCRP^[152]^, and it has been considered a good lead compound in the search for new MDR inhibitors^[153]^.

The deletion of the tetrahydroisoquinoline group of tariquidar (59) led to compounds that were shown to be selective BCRP inhibitors^[152-154]^ [Figure 11]. In 2015, Marighetti *et al.*^[155]^ reported a quantitative structure-activity relationship (QSAR) study of this class of BCRP inhibitors. The inhibitory activities against BCRP and P-gp were determined on BCRP-overexpressing MCF-7 MX cells, by a Hoechst 33342 assay, and on A2780 adr cells by a calcein AM assay, respectively.

By the comparison between activity and calculated logP values a tendency of higher activity with greater lipophilicity was observed. In fact, the most BCRP active compounds 60 and 61 are the most lipophilic. Compound 60 showed high and selective activity for BCRP (IC_50_ = 0.94 M) since no P-gp inhibitory activity was observed. Otherwise, compound 61 was active also on P-gp despite with lower potency (IC_50_ = 0.56 M on BCRP and 6.07 M on P-gp)^[155]^.

**Figure 12:**
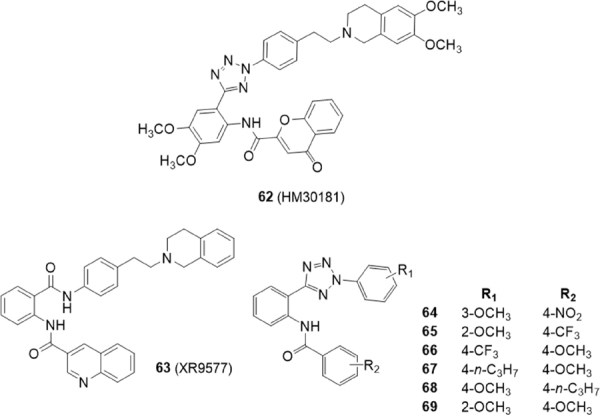
HM30181 derivatives

Compound 62 (HM30181) has been recently identified as a third-generation P-gp inhibitor^[156]^ [Figure 12]. This compound is a tetrazole derivative structurally related to tariquidar (59) with a tetrazole ring instead of an amide linker.

In 2015, Köhler *et al.*^[157]^ reported 21 tariquidar derivatives lacking the tetrahydroisoquinoline moiety and, as compound 62, an amide linker was replaced by a tetrazole ring [Figure 12]. The inhibitory activity against BCRP was evaluated by the Hoechst 33342 accumulation assay using the MDCK II BCRP cell line. The activity on P-gp and MRP1 was investigated using P-gp overexpressing cell line A2780 adr and MRP1 overexpressing cell line H69AR, respectively, by the calcein AM assay. These compounds lack methoxy groups at their middle phenyl ring as the reference compound XR9577(63). In general, electron withdrawing groups on the benzamide moiety (R_2_) led to inactive compounds or BCRP inhibitors showing a decreased maximal response [*I*max: maximum response of tested compounds in relation to elacridar (63)]; as an example, compound 64 (R_2_ = 4-NO_2_) was found to be a potent BCRP inhibitor with an IC_50_ value of 0.0789 μM but showed an *I*max value of 58. However, compound 65 (R_2_ = 4-CF_3_) with an IC_50_ value of 0.451 M showed a maximal response comparable to that of elacridar (63) and Ko143 (5) (*I*max = 100)^[157]^.

The most potent and selective compounds 66, 67 and 68 bearing electron donating groups on the benzamide ring (R_2_) showed about 2-fold higher BCRP inhibitory activities than Ko143(5) (IC_50_ value of 0.0642 μM, 0.0794 μM, 0.0730 μM and 0.128 μM, respectively).

Compounds 65 and 69 interact with both BCRP and P-gp in fact, a 2-methoxygroup on phenyl tetrazole moiety (R_1_) leads to a moderate inhibitory activity on P-gp lowering BCRP selectivity (65, IC_50_ = 11.0 μM on P-gp, IC_50_ = 0.451 μM on BCRP; 69, IC_50_ = 7.73 μM on P-gp, IC_50_ = 0.180 μM on BCRP)^[157]^.

**Figure 13:**
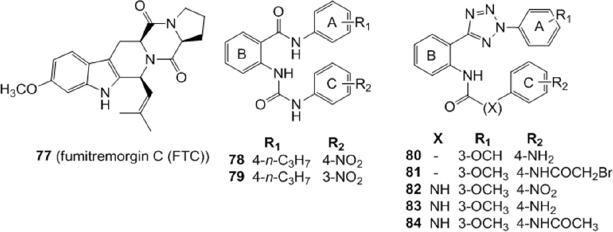
Tetrazole derivatives

In 2016, Köhler *et al.*^[158]^ studied another series of phenyltetrazolylphenylamides on BCRP to determine the specific influence of different substituents at the two external phenyl rings (A and C) [Figure 13]. BCRP-overexpressing MDCK II BCRP cell line was used to evaluate the inhibitory effect on BCRP by the Hoechst 33342 or pheophorbide A accumulation assay.

The activity on P-gp and MRP1 was investigated by the calcein AM assay using P-gp overexpressing cell line A2780 adr and MRP1 overexpressing cell line H69AR, respectively.

The most potent compound on BCRP was the unsubstituted derivative 70 with IC_50_ values three to four times lower than Ko143 (5) in the Hoechst 33342 accumulation assay or the pheophorbide A accumulation assay, respectively [70, IC_50_ = 0.0669 and 0.0640 μM, Ko143(5), IC_50_ = 0.221 and 0.276 μM]. A few compounds showed moderate activities on P-gp but, interestingly, compound 71 showed a threefold higher inhibitory activity toward P-gp (IC_50_ = 0.434 μM) than cyclosporine A (IC_50_ = 1.21 M). The good P-gp activity of this hydroxyl derivative is rather surprising since usually hydroxyl substituted compounds are poorer P-gp inhibitors than their methoxy counterparts, due to reduced lipophilicity. Compound 71 is a good dual inhibitor against P-gp and BCRP achieving a BCRP- inhibiting potency like Ko143 5 in the Hoechst 33342 accumulation assay [71, IC_50_ = 0.204, Ko143(5), IC_50_ = 0.221]. An investigation on the type of interaction with the BCRP substrates Hoechst 33342 and pheophorbide A of compound 72 and Ko143(5) revealed that both compounds showed a competitive interaction with the substrate Hoechst 33342. On the contrary they acted as non-competitive modulators in presence of pheophorbide A; this result suggests that these two compounds bind to a different site of the BCRP protein with respect to pheophorbide A.

Two years later Köhler *et al.*^[159]^ continued the study on structure-activity relationships of phenyltetrazolylphenylamides derived from tariquidar for BCRP inhibition. Using the unsubstituted *N*-(2-(2-phenyl-2*H*-tetrazol-5-yl)phenyl)-benzamide 70^[158]^ as lead structure, 38 new derivatives with several modifications of the three phenyl rings (A, B, C) have been synthesized. The 2,5-disubsituted tetrazole moiety was exchanged for bioisosteric structures like 2,5-disubstituted 1,3,4-oxadiazole and an 1,4-disubstituted 1,2,3-1*H*-triazole. The ortho amide linker was modified and meta and para positioned linkers (inverted amide, sulfonamide, tetrazole) were inserted or the amide linker was removed. Moreover, either ring A and C were modified.

The study of these compounds on the MDCK II BCRP cell line, using the Hoechst 33342 accumulation assay, revealed that for BCRP a bioisosteric substitution of the tetrazole moiety is not beneficial as well as the modification of the amide in ortho-position. The ring A is essential for BCRP activity since its elimination resulted in inactivity. The introduction of a methoxy group at phenyl ring B exerted a slightly positive effect on the activity since compound 74 showed a similar activity than the corresponding analogue without that substituent (75), previously reported (IC_50_ = 0.0774 and 0.106 M, respectively)^[157]^.

The modification of the phenyl A in a pyridyl moiety along with the presence of methoxy groups in *para*-position of both rings A and C improved inhibitory potency; in fact, compound 76, the most potent of the series, showed an IC_50_ value of 0.0616 M while the corresponding analogue with a phenyl ring in A (73) showed an IC_50_ value of 0.199 M^[158]^. Most of the compounds exhibited low activities against P-gp in the calcein AM assays using P-gp overexpressing A2780 adr cell lines which therefore appeared to be selective inhibitors.

Compounds 60^[155]^ and 64^[157]^ have been used as leads by Gujarati *et al.*^[160]^ to synthesize several benzamide and phenyltetrazole derivatives with amide and urea linkers between rings B and C [Figure 13]. Various substituents were introduced on rings A and C to explore the consequences of steric, electronic and solubility characteristics on BCRP activity. Several compounds exhibited reversal effects comparable to the known BCRP inhibitor fumitremorgin C (FTC) (77) on the cytotoxicity of mitoxantrone in BCRP-overexpressing H460/MX20 cells at three different concentrations (1, 3, 10 M). Fold resistance was calculated as ratio between the IC_50_ values of the substrate (MX) in presence or absence of inhibitor by the IC_50_ of parental cells without inhibitors. In the benzamide series the most potent compounds possessing a urea linker were 78 and 79 with fold resistances of 1.51 and 1.62 respectively, at 10 M concentration, proving to be more potent than the lead compound 60 (fold resistance of 2.76). Compounds 80 and 81, with an amide linker, and 82, 83 and 84, with an urea linker, were the most potent analogues in the tetrazole derivatives with fold resistances of 1.39, 1.32, 1.87, 1.76 and 1.51 respectively, at 10 M concentration; these values were comparable to that of 64 (1.43), the lead compound of the tetrazole analogues with an amide linker. The corresponding value of FTC (77) [Figure 13] is 1.49. These low cytotoxic compounds appeared to be selective BCRP inhibitors since they did not show any reversal effect on paclitaxel resistant P-gp overexpressing SW620/Ad300 cells. Compounds 78, 79, and 80 were found to be stimulators of basal ATPase, while compound 82 was found to be an ATPase inhibitor^[160]^.

**Figure 14:**
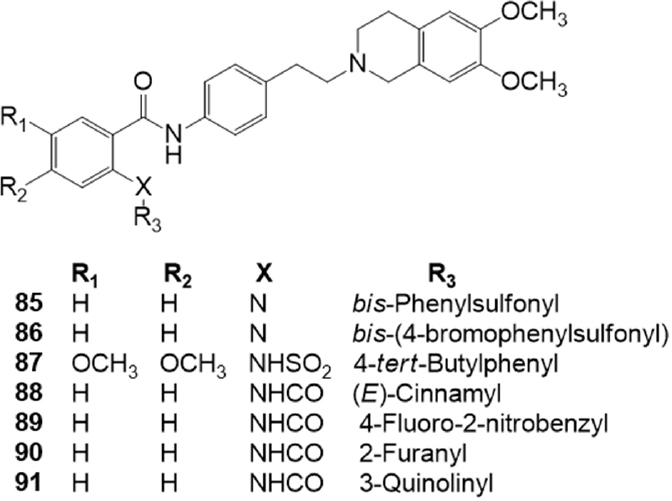
Sulfonamido derivatives of tariquidar

A series of amide or sulfonamide derivatives of tariquidar were studied by Li *et al.*^[161]^ in 2015 [Figure 14]. The compounds were tested for their activity in the inhibition of Rh123 efflux in P-gp transfected cell line (K562/P-gp cells), or of pheophorbide A efflux in BCRP transfected cell lines (K562/BCRP cells). Their cytotoxicity was evaluated in normal human colon fibroblasts (CCD18-Co), human gastric epithelial cell line and primary rat liver cells. These 6,7-dimethoxytetrahydroisoquinoline derivatives were modified on the anthranilic acid amide which was decorated with different functional groups (aromatic, heterocyclic or chain structures). A sulfonamide group was introduced instead of an amide function to improve aqueous solubility and this modification led to specific P-gp inhibitors. In fact, the low toxic and aqueous soluble sulfonamide derivatives 85, 86 and 87, were found to specifically inhibit the Rh123 efflux in P-gp transfected cell lines (K562/P-gp). The amide derivatives 88, 89, 90 and 91 were instead found to dually inhibit P-gp and BCRP-mediated drug efflux in P-gp or BCRP transfected cell lines (K562/P-gp and in K562/BCRP cells, respectively) and their cytotoxicity was much lower than tariquidar (59). The P-gp and BCRP inhibition by these tariquidar derivatives was found to be associated with the inhibition of ATP hydrolysis.

**Figure 15:**
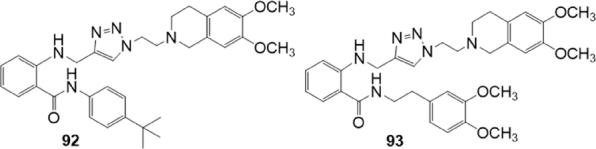
Triazole derivatives

In 2018, Gao *et al.*^[162]^ investigated 17 derivatives structurally related to tariquidar 59 as P-gp inhibitors [Figure 15]. An amino metil-1,2,3-triazole moiety was inserted instead of the phenylamido group and the second amide of the tariquidar (59) structure was inverted. The effect of these compounds on reversing adriamycin resistance towards P-gp overexpressing cell line (K562/A02) was investigated by MTT method. Structure-activity relationship studies suggested that the presence of a 4-*tert*-butylphenyl moiety on the inverted amide (compound 92), conferred the best P-gp inhibitory activity (IC_50_ = 1.22 M) with a reversal fold (RF) of 39 [RF = (adriamycin IC_50_ value without modulator)/(adriamycin IC_50_ value with 5 M modulator)], which was close to that of tariquidar (59) (RF = 49.4). On the contrary, the presence of a 3,4-dimethoxyphenethyl group led to a relatively ineffective compound (93, IC_50_ = 41.48 M). Compound 92 showed a 24 h duration lasting, was endowed with low cytotoxic and able to inhibit the efflux of Rh123 in K562/A02.

**Figure 16:**
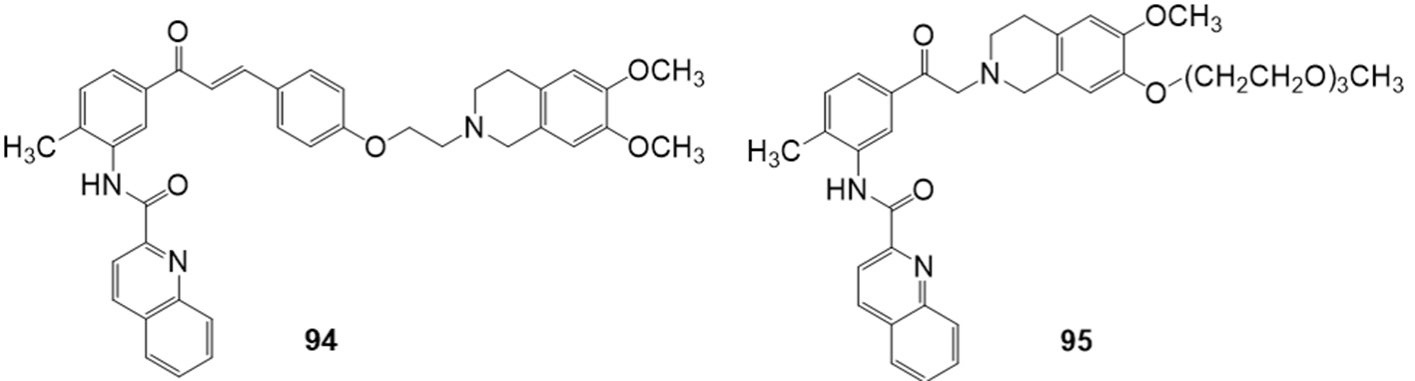
Chalcone and ketone derivatives of tariquidar

In search for more stable analogues, Peña-Solórzano *et al.*^[163]^ prepared a series of tariquidar analogues with chalcone and ketone moieties, according to a bioisosteric approach [Figure 16]. These compounds were investigated for their inhibitory activity and selectivity toward BCRP. The most potent compounds of the two series have a methyl group at the amine chalcone core suggesting the importance of this group in this position. In the chalcones series, correlation between potency and lipophilicity was found, in fact, the most potent compounds showed the highest calculated partition coefficient values. Compound 94, with the highest clogP value (6.97), showed an IC_50_ value of 0.88 μM in the Hoechst assay on BCRP-overexpressing MCF-7/Topo cells, that is similar to that of reference compound fumitremorgin C (FTC) (77) (IC_50_ = 0.731 μM). The modulating activities for P-gp and MRP1 were evaluated by calcein assay using Kb-V1 (P-gp) and MDCK II (MRP1) cell lines, respectively. The results suggested that the chalcones series had low inhibitory activity on P-gp and MRP1 both at 1 μM and 10 μM. Ketone series exhibited lower BCRP modulating activity than the chalcone series. The most potent compound of the series, 95 showed an IC_50_ value of 6.65 μM in the Hoechst assay with a small preference for BCRP over P-gp and MRP1, evaluated by calcein assay using Kb-V1 (P-gp) and MDCK II (MRP1) cell lines. These results indicated that the lipophilicity plays a critical role for the BCRP modulation and selectivity when comparing the two series of compounds. Compound 94 showed a concentration dependent cytostatic effect on proliferating MCF-7/Topo cells that could be ascribed to the reactive α−β-unsaturated ketone core. The ketone derivative 95, which is lacking the Michael acceptor system, showed indeed a reduced cytotoxicity^[163]^.

**Figure 17:**
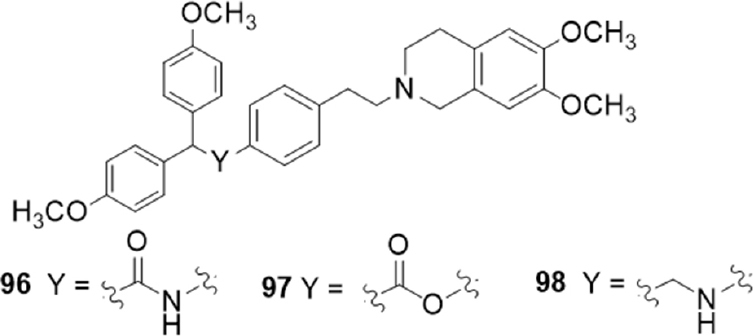
Aryl-substituted derivatives of tariquidar

Tariquidar derivatives with the 6,7-dimethoxy-2-phenethyl-1,2,3,4-tetrahydroisoquinoline group connected to aryl-substituted moieties by amide, ester and alkylamine functions, were investigated for their P-gp, MRP1 and BCRP activity^[164]^ [Figure 17].

Compounds containing an amide or alkylamine group generally displayed higher activity values than the corresponding ester derivatives in the calcein-AM test on P-gp overexpressing cells (MDCK-MDR1 cells). However, compounds bearing a 2,2-bis (4-methoxyphenyl) moiety as aryl residue showed the best P-gp activity in all three series with comparable values (96, EC_50_ = 0.30 μM; 97, EC_50_ = 0.33 μM; 98, EC_50_ = 0.57 μM). Amide and alkylamine derivatives showed no activity toward both MRP1 and BCRP evaluated by measuring the inhibition of the efflux of calcein-AM in MRP1 overexpressing cells (MDCK-MRP1 cells) and of Hoechst 33342 in BCRP overexpressing cells (MDCK-BCRP cells), respectively. Unusually, the substitution of the amide function with the ester one led to derivatives that showed low activities on BCRP (EC_50_ values between 5.9 and 17 μM).

All compounds were stable to enzymatic hydrolysis evaluated in human plasma, even those containing an amide or ester group not impairing, therefore, the *in vivo* bioavailability^[164]^.

## Others

Beside the compounds described previously, other compounds belonging to different chemical classes have been described, both as BCRP selective modulators and as P-gp/BCRP dual modulators.

### Tetrahydro-β-carbolines

**Figure 18:**
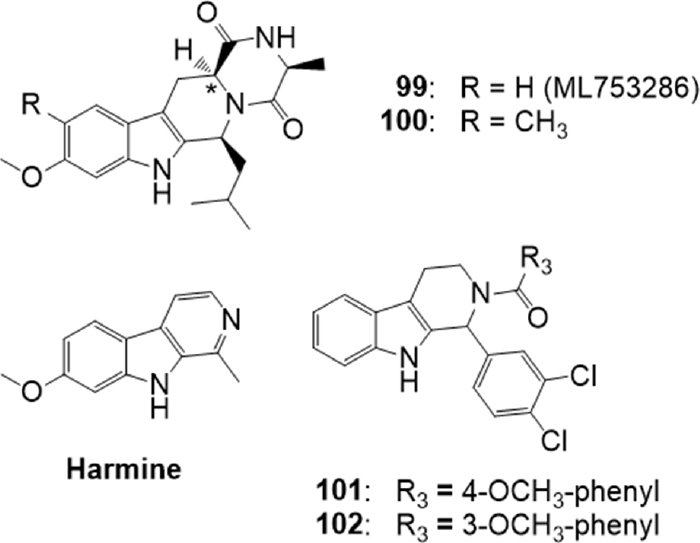
Tetrahydro-β-carboline derivatives

Some derivatives were designed based on Ko143 structure, which presents a tetrahydro-β-carbolinic scaffold. Since Ko143(5) exhibits an unfavorable pharmacokinetic profile, probably due to hydrolysis of the ester moiety, Li *et al*.^[165]^ designed and synthesized some Ko143 derivatives, to explore the structure-activity relationships in this field and to obtain new compounds with a better PK profile. As first, three desmethoxy analogs were prepared; moreover, a series of compounds devoid of the ester group and showing different stereochemistry were obtained [Figure 18]. To determine the inhibitory potency of the new compounds, the IC_50_s of these derivatives on BCRP mediated E3S (estrone sulfate) transport were measured on Caco-2 cells. Results suggested that the ester portion of the lead compound Ko143 is not necessary for the activity and can be replaced by more stable and simple groups, such as a methyl group. In fact, compounds 99 and 100 showed almost the same potency as that of Ko143 (IC_50_ of Ko143 was determined along with each synthesized compound because of the variability in the determination of the parameter); the diastereoisomers, which present a different configuration at the marked stereocenter [Figure 18], failed to exhibit activity up to 10 M. The potency of 99 on P-gp inhibition was also evaluated on Caco-2 cells but, as the lead compound Ko143, it showed an IC_50_ > 30 M, behaving as selective BCRP inhibitor. Both 99 and 100 showed also a better PK profile in rats with respect to Ko143^[165]^.

On 99, re-named ML753286, further tests were conducted^[166]^ to characterize the preclinical properties. ML753286 confirmed to be a selective inhibitor for BCRP, without any effect not only on P-gp, but also on organic anion transporting polypeptide and major cytochrome P450s. The compound did not behave as efflux transporter substrate and showed good *in vitro* ADME properties. *In vivo* PK experiments in mouse and rat confirmed its *in vitro* potency and identified the optimal dose of ML753286 (50-300 mg/Kg orally) to inhibit Bcrp in rodents^[166]^.

Also the alkaloid harmine [Figure 18] was reported to be an BCRP inhibitor^[167]^. This compound contains the β-carboline moiety, a substructure like that presented by BCRP inhibitors Ko143(5) and FTC (77). On this basis, Wiese *et al*.^[168]^ synthesized 37 new tetrahydro-β-carboline derivatives with a tricyclic common scaffold and different substituents on the phenyl ring, on the indole nitrogen N-9 and on the N-2 present in the saturated ring. All synthesized compounds were tested for their inhibitory potency toward BCRP using the Hoechst 33342 accumulation assay with Madin-Darby canine kidney II (MDCK II) cells overexpressing the transporter. Some selected compounds were additionally tested in the pheophorbide A assay: the comparison of the pIC50 values obtained in both assays showed a very high correlation. Many compounds were active, and derivatives 101 and 102 displayed the best activity (IC50 = 0.233 M and IC50 = 0.238 M respectively in the Hoechst 33342 assay). SAR suggested that the hydrogen bond donor-acceptor system of the hydrogen at indole N9 and the acyl group at N2 were necessary for the inhibitory activity. Moreover, chlorine or bromine substitution in meta and/or para position at the phenyl ring gave highly potent compounds with an inhibitory activity in the submicromolar range. The pharmacological profile of the two compounds and of some analogs was deepened by evaluating their toxicity, their ability to reverse MDR and their inhibition on ATPase activity^[168]^. Cytotoxicity data show that their inhibitory effect is substantially higher than their toxicity, and results confirmed that they are able to reverse the BCRP-mediated resistance toward SN-38 and to inhibit the ATPase activity. All compounds were also screened for ABCB1 inhibition to investigate their selectivity toward BCRP, using the calcein AM assay with the A2780 adr cell line, but most of them showed just a slight effect on ABCB1 at 10 μM. The most potent compounds included 101 and 102 showed to be selective BCRP inhibitors.

### Other heterocycles and cyclic compounds

Other heterocycle derivatives were tested as BCRP inhibitors.

**Figure 19:**
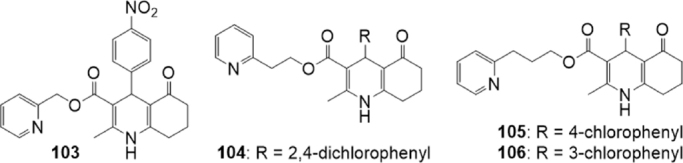
5-Oxo-hexahydroquinoline derivatives

Ranjbar *et al*.^[169]^ described a series of 5-oxo-hexahydroquinolines with pyridyl methyl carboxylate substitutions such as compound 103 [Figure 19] which behaved as P-gp inhibitors.

Based on these results, very recently the same group^[170]^ synthesized twelve 5-oxo-hexahydroquinolines bearing different aromatic substitutions at C4 while having 2-pyridyl alkyl carboxylate substituents (ethyl or propyl), on the basis of preliminary drug-likeness properties calculation that indicated that all the proposed compounds could be successful oral drug candidates. The inhibitory effects of the compounds on P-gp, MRP1 and BCRP were evaluated by flow cytometric determination of accumulation of rhodamine 123 in, respectively, P-gp-overexpressing MES-SA/DX5 cells, calcein AM in MRP1-overexpressing Flp-In HEK293 cells and mitoxantrone in BCRP-overexpressing HEK293 cells. To confirm the P-gp inhibitory activity, the effect of compounds on the reduction of doxorubicin’s IC_50_ on drug-resistant human uterine sarcoma cell line, MES-SA/DX5, was also evaluated^[170]^.

Most of the compounds showed significant inhibition of P-gp transporter and two of them showed an outstanding activity in the doxorubicin resistance reversal. Some of the derivatives were also interesting MRP1 inhibitors, and some of them were efficacious in inhibiting BCRP. Analogs containing chlorine moiety on the phenyl ring, including 104, 105 and 106 [Figure 19] showed multiple modulatory activity being active on both P-gp and BCRP, but also on MRP1 transporter, in inhibiting transporter-mediated efflux of the probe. None of the compounds showed to be a selective inhibitor of BCRP. Interestingly, some of the compounds were able to induce collateral sensitivity, but this effect was MRP1-dependent^[170]^.

**Figure 20:**
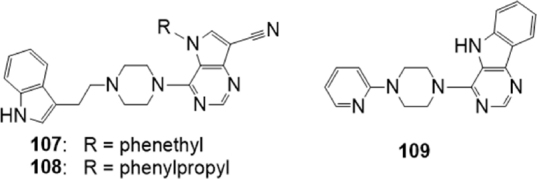
9-Deazapurine derivatives

Also a series of more than 30 compounds bearing the 9-deazapurine scaffold [Figure 20] were synthesized^[171]^, since it is a substructure present in pyrrolopyrimidines^[172]^ and indolopyrimidines^[173]^ which showed to be transporter inhibitors. Their inhibitory activity on P-gp, MRP1 and BCRP was measured: for P-gp, calcein AM efflux was evaluated in P-gp overexpressing A2780 adr cell line; for MRP1 the daunorubicin assay on H69AR overexpressing MRP1 cells (ATCC CRL-11351) was used; finally, the effect on BCRP was evaluated by the pheophorbide A assay on MDCK II BCRP cells which overexpress this protein.

The obtained 9-deazapurines were classified according to their structure into four classes: compounds belonging to the first three classes were in most cases MRP1 inhibitors or dual MRP1/P-gp inhibitors, showing IC_50_s in the micromolar or submicromolar range for the latter transporter. However, two compounds belonging to the second class, 107 and 108 [Figure 20], behaved as promising inhibitors toward the three transporters, being active in the low micromolar or in the nanomolar range (IC_50_ on P-gp, MRP1 and BCRP 1.64 M, 0.524 M and 1.39 M respectively for 107 and 1.46 M, 0.501 M and 1.69 M respectively for 108). In this class, a relationship between side chain length and biological activity in the case of P-gp and BCRP could be observed.

The fourth class was constituted by 8,9-annulated 9-deazapurines. This class showed high affinity in respect to BCRP, with different effects in respect to P-gp and MRP1. The most interesting compound was 109 [Figure 20], a good inhibitor of BCRP but also of P-gp and MRP1 (IC_50_ of 1.81 μM, 5.00 μM and 0.495 μM respectively). Compounds 107, 108 and 109 are triple inhibitors with IC_50_s in the single-digit micromolar range. Intrinsic toxicity of 108 was further investigated: this derivative showed no or little toxic effect on resistant cell lines overexpressing the three transporters and on the corresponding sensitive cells. Moreover, compound 108 behaved as noncompetitive inhibitor on the three transporters, and was able to restore sensitivity to daunorubicin (P-gp and MRP1) and SN-38 (BCRP) in resistant cells, although only in part in the case of P-gp-overexpressing ones^[171]^.

**Figure 21:**
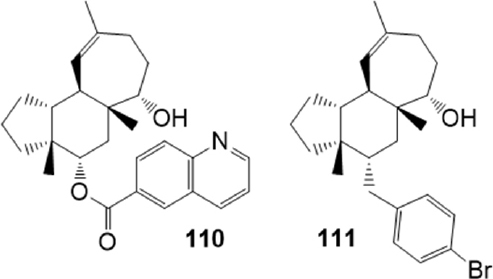
Homoverrucosanoid derivatives

As stated, a promising w. ay to identify new lead compounds that modulate MDR is the study of natural compounds^[174]^ as macrocyclic diterpenoids. Schäfer *et al*.^[175]^ described the total synthesis of simplified analogs of gagunin E, a marine homoverrucosanoid isolated in 2002 from a sponge of the genus *Phorbas*^[176]^. A library of 16 of non-natural homoverrucosanoid-(cyclohepta[*e*]hydrindanoids) derived esters [Figure 21] were examined as inhibitors of the membrane transporter proteins ABCB1, ABCG2 and ABCC1, using the calcein AM accumulation assay on both ABCB1 overexpressing A2780 adr cells and ABCC1 overexpressing H69AR cells, and the Hoechst 33342 accumulation assay using the ABCG2 overexpressing MDCK II ABCG2 cell line respectively.

SAR studies revealed that specific structure, configuration and general lipophilicity of the homoverrucosane scaffold are important factors for effective molecular recognition. The presence of an S-configured alcohol at C-6 and a quinoline-6-carboxylic acid ester at C-4 were beneficial for binding to ABCG2 and ABCB1: the quinolinecarboxylate 110 (IC_50_ = 1.56 M on ABCG2 and IC_50_ = 1.39 M on ABCB1) represented the most potent dual ABCG2/P-gp modulator of the collection; the bromobenzoate 111 instead showed a selective profile, exhibiting a strong inhibitory effect on ABCG2 (IC_50_ = 2.91 M) without affecting ABCB1-mediated efflux, and a low cytotoxicity.

### Propafenone derivatives

**Figure 22:**
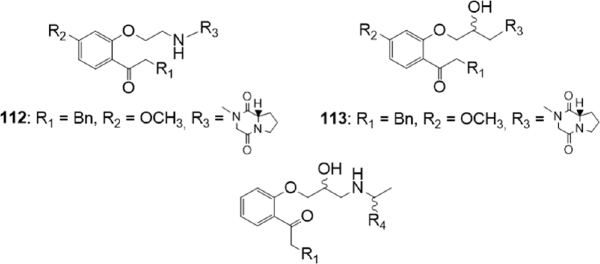
Propafenone derivatives

Continuing a previous research on propafenone derivatives^[177]^, Schwarz *et al*.^[178]^ synthesized novel propafenones aiming to define the molecular characteristics eliciting transporter selectivity [Figure 22].

The design of the new derivatives took into consideration two molecular characteristics: the presence of a basic nitrogen atom and the presence of a rigid moiety on the chain, since a non ionizable nitrogen atom, the presence of rotatable bonds and the presence of H-bond acceptors could influence both activity and selectivity. The nature of the spacer connecting the nitrogen atom and the central aromatic ring was modified, the flexibility of substituent R_3_ [Figure 22] was modified by inserting a proline or a diketopiperazine moiety, the lipophilicity was varied by inserting substituents on different position, and the effect of basicity of the nitrogen atom was evaluated synthesizing some aliphatic *N*-substituted analogues, as described by the general structures.

The inhibitory activity on P-gp and BCRP was evaluated, measuring the inhibition of the intracellular accumulation of the substrates daunorubicin (P-gp overexpressing cells) or mitoxantrone (BCRP overexpressing cells).

Some of the tested compounds were active as inhibitors of both transporters; only the methoxy substituted compounds 112 (IC_50_ = 2.3 M on BCRP and IC_50_ = 6.2 M on P-gp) and 113 (IC_50_ = 3.8 M on BCRP and IC_50_ = 11.0 M on P-gp) [Figure 22] carrying a piperazinedione residue were more active on BCRP than on P-gp.

In the meantime, to predict the inhibition against BCRP and P-gp for the designed compounds, two in silico classification models were created. In addition, the logP values of all compounds were calculated. Results of the theoretical models were compared with the experimental results. The BCRP- and the P-gp-inhibition model showed different trends; results indicated that both the flexibility of the substituent at the nitrogen atom, and the basicity of the nitrogen atom, influenced transporter selectivity. Moreover, logP values seemed to have a higher influence on the inhibitory activity on P-gp with respect to that on BCRP.

### Amide or ester derivatives

**Figure 23:**
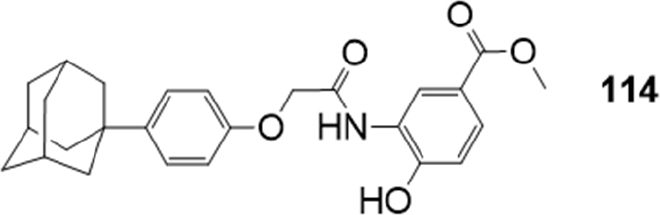
HIF-1 inhibitor

Kim *et al*.^[179]^ synthesized compound 114 [Figure 23], an inhibitor of the hypoxia inducible factor-1 alpha (HIF-1), which plays an important role in angiogenesis and metastasis and is a promising therapeutic target for the development of anticancer drugs. Additional studies suggested that the compound inhibited HIF-1 stability via direct binding with calcineurin b homologous protein 1 (CHP1).

Recently, the effects of 114 on the functional activity and gene expression of two major efflux transporters, BCRP and P-gp were evaluated^[180]^, by using MDCKII cells overexpressing each transporter (MDCKII-BCRP and MDCKII-MDR1). Its effects on the cytotoxicity of co-administered mitoxantrone and doxorubicin were also evaluated in both MDCKII-mock and MDCKII-BCRP transfected cells: the derivative down-regulated BCRP expression and made resistant cells more susceptible to the cytotoxicity of the anticancer drugs. Furthermore, 114 improved the oral exposure of methotrexate by twofold in rats. In contrast to BCRP, the compound had no inhibition effect on cellular accumulation of P-gp substrate rhodamine 123 and gene expression of P-gp, showing to behave as potent and selective BCRP inhibitor.

**Figure 24:**
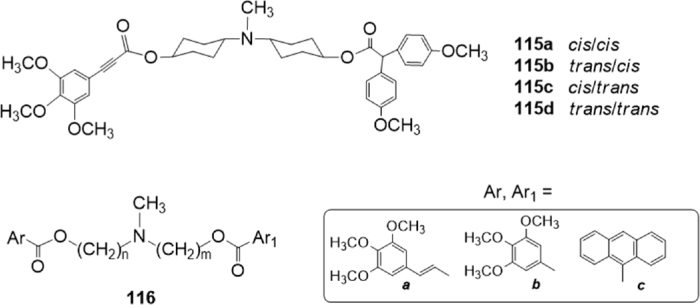
Ester derivative

Continuing their previous researches on derivatives of the natural alkaloid Pervilleine A, and based on the polyvalency approach, Teodori *et al*.^[181]^ synthesized a set of basic molecules carrying aryl moieties connected to the *N*-containing linker through ester functions. The nature of the spacer (dicyclohexylamine or dialkylamine) and of the aryl moieties was modified to investigate their interacting mechanism with the ABC transporters and their selectivity [Figure 24]. To prevent the possible metabolic instability of the ester functions, a little number of derivatives containing the amide group were also studied.

The inhibitory effect on P-gp, MRP1 and BCRP of the synthesized molecules was evaluated, measuring the inhibition of the efflux of Calcein-AM in P-gp overexpressing MDCK-MDR1 cells and in MRP1-overexpressing MDCK-MRP1 cells, and of Hoechst 33342 in BCRP-overexpressing MDCK-BCRP cells. All compounds were also tested to evaluate their intrinsic cytotoxicity at 1 M. P-gp interacting-mechanism of the derivatives was further investigated by measuring Apparent Permeability (*P*_*app*_) (BA/AB) in Caco-2 cell monolayer and ATP cell depletion in MDCK-MDR1cells. All dicyclohexylamine derivatives were found to be P-gp substrates, whereas a dialkylamine derivative was a P-gp inhibitor. Replacement of the ester function with the amide one led to compounds that are inactive or poorly active on the transporters.

Among the obtained compounds, the four bis(cyclohexanol)amine geometrical isomers 115^[182]^ [Figure 24] bearing trans-3,4,5-trimethoxycinnamyl and 2,2-bis-(4-methoxyphenyl) moieties are particularly interesting because of their clear-cut dependence on stereochemistry for their potency. Among these four geometric isomers, the highest potency was found for the *trans/cis* isomer 115b with activity in the nanomolar range (EC_50_ = 1.2 nM). All four isomers were inactive on MRP1 but were active on BCRP (EC_50_ in the single-digit micromolar range except for the *cis/cis* isomer).

These results suggested that the nature and the stereochemistry of the spacer influence the interaction binding mode of these derivatives. Moreover, the nature of the aryl moieties influences the selectivity since the set of isomers 115 are P-gp/BCRP modulators inhibitors.

Recently, based on the same approach, the same research group^[183]^ synthesized several *N,N*-bis(alkanol)amine aryl esters characterized by the presence of a basic nitrogen atom linked to two different aromatic ester portions by two polymethylenic chains of variable length as spacers, described by the general structure 116 [Figure 24].

Keeping in mind preceding results^[184-186]^, they synthesized all the possible isomers bearing a combination of chains of variable length, for a total length of 8, 9 and 10 methylenes, and carrying different aromatic residues. P-gp inhibiting activity was evaluated on K562/DOX doxorubicin resistant cells, measuring their effect on THP-adriamycin (pirarubicin) nuclear uptake. Results of this preliminary assay indicated that derivatives showing a total spacer of 10 methylenes were always very active, regardless of the combination of aromatic residues and of *n* and *m* chain lengths. Chemical stability both in phosphate buffer solution and in human plasma was evaluated, indicating that most of the obtained molecules were stable in both matrices.

Selected molecules were further tested evaluating their effect on rhodamine 123 assay in the resistant K562/DOX cell line. Moreover, the inhibitory effect on P-gp, MRP1 and BCRP was evaluated, measuring the inhibition of the efflux of Calcein-AM in P-gp overexpressing MDCK-MDR1 cells and in MRP1-overexpressing MDCK-MRP1 cells, and of Hoechst 33342 in BCRP-overexpressing MDCK-BCRP cells. In these tests, the molecules maintained a good activity on P-gp (EC_50_ comprised between 0.20 and 0.92 M) and good or moderate activities on BCRP (EC_50_ comprised between 3.0 and 16.0 M). At the contrary, only derivatives carrying a 3,4,5-trimethoxyphenyl residue [labelled b in Figure 24] together with the trans-3,4,5-trimethoxycinnamyl one [labelled a in Figure 24] are active on MRP1, while the other compounds showed no activity in this test. Therefore, this finding suggests that suitable modification of the aromatic moiety can address selectivity among the transporters in this series of molecules. P-gp interacting-mechanism was investigated by measuring Apparent Permeability (*P*_*app*_) in Caco-2 cell monolayer in combination with ATP cell depletion in MDCK-MDR1 cells: two molecules resulted as unambiguous substrates whereas the other compounds behaved as not-transported substrates^[187]^.

### Tyrosine kinase inhibitors

Tyrosine kinase inhibitors (TKIs) are a group of target-specific anticancer drugs. Gefitinib (45) [Figure 10], a representative molecule of this class, was described previously as lead compound. Unfortunately, increasing resistance to TKIs has been reported, and the enhanced efflux of these derivatives by overexpression of ABCB1 and ABCG2 has been considered as responsible for the resistance. Further studies have indicated that many TKIs behave as dual substrates of P-gp and BCRP or behave as inhibitors. Therefore, TKIs can also act as MDR modulators based on their effect on the transporters. Evaluation of this series of compounds is beyond the aim of this review, but some recent papers describing the inhibition of BCRP or P-gp/BCRP transporters by TKIs are reported^[188-191]^.

## Conclusions

MDR is a kind of acquired drug resistance to anticancer drugs. This phenomenon is often related to the overexpression of membrane proteins belonging to the ABC protein family, such as P-gp (ABCB1), BCRP (ABCG2) and MRP1 (ABCC1), which behave as efflux transporters. P-gp is the first transporter discovered to be involved in cancer drug resistance, and over the years, inhibitors of this pump have been disclosed to administer them in combination with chemotherapeutic agents. Three generations of inhibitors of P-gp have been examined in preclinical and clinical studies; however, these trials have failed to demonstrate an improvement in therapeutic efficacy of the co-administered antitumor agent. Nevertheless, the concept that modulation of ABC efflux transporters may overcome MDR is still strong, and the search for new chemosensitizers is still ongoing. Moreover, an intriguing aspect that emerged from the studies was that the ABC transporters P-gp, MRPs and BCRP are often co-expressed in tumors and that they have an overlapped specificity for a variety of substrates. Therefore, selective inhibition of one efflux transporter could be compensated by the remaining transporters, and in the last years many studies were devoted to evaluating the inhibitory activity of new derivatives on cells overexpressing ABCB1, ABCC1 and ABCG2 respectively.

In the last two decades, after the discovery of BCRP, many new molecules acting as BCRP-dependent MDR modulators were reported. BCRP and P-gp are overexpressed together in several haematological and solid tumours. Moreover, P-gp and BCRP are the two main ABC transporters placed at the BBB and reduce the ability to cross the BBB of many substrates of these two proteins including chemotherapeutic agents.

On this basis, both selective BCRP inhibitors and dual P-gp/BCRP ones are needed to define sound SARs and to discover new leads for the development of new molecules. As described in the review, many research groups are involved in this field, and many new molecules were reported in the last five years in the literature. Some of them could be promising starting points for the design of new compounds.

## Declarations

### Copyright

© The Author(s) 2019.
